# Validation of phiC31-mediated expression and functional knockout of Opn3 in the *Opn3-phiC31o* knock-in mouse

**DOI:** 10.1186/s40662-025-00455-z

**Published:** 2025-10-17

**Authors:** Rachel Kam Yan Kwok, Hikaru Ikuta, Chisato Iba, Yuka Nakano, Ziyan Ma, Yahan Chuai, Yuichi Hiraoka, Taichi Sayanagi, Toshihide Kurihara, Satoru Moritoh, Kenji F. Tanaka

**Affiliations:** 1 Division of Brain Sciences, Institute for Advanced Medical Research, Keio University School of Medicine, 35 Shinanomachi, Shinjuku-Ku, Tokyo 160-8582 Japan; 2 Laboratory of Photobiology, Keio University School of Medicine, Shinjuku-Ku, Tokyo 160-8582 Japan; 3 Laboratory of Animal Resources, Center for Disease Biology and Integrative Medicine, Graduate School of Medicine, University of Tokyo, 7-3-1 Hongo, Bunkyo-Ku, Tokyo 113-0033 Japan; 4 Department of Neurosurgery, Keio University School of Medicine, 35 Shinanomachi, Shinjuku-Ku, Tokyo 160-8582 Japan

**Keywords:** Non-visual opsins, Opn3, Mouse, Brain, Retina, phiC31o

## Abstract

**Background:**

Opn3 is a non-visual blue light-sensitive opsin that has recently been reported to have an expansive repertoire of biological functions. To investigate the function of Opn3-expressing cells, we aimed to generate a system in which Opn3-expressing cells can be targeted by site-specific gene recombination.

**Methods:**

*Opn3-phiC31o* knock-in (KI) mice were generated using the CRISPR-Cas9 method. The *phiC31o-poly(A)* cassette was inserted into the translation start site in exon 1. *Opn3* mRNA and *phiC31o* mRNA were visualized by in situ hybridization (ISH). 5' rapid amplification of cDNA end (5' RACE) analysis was performed using RNAs from wild-type mouse cerebral cortex and cerebellum to identify the transcription start site of *Chml*, predicted to be shared with the transcription start site of *Opn3*. Cold-induced decrease in body temperature was monitored with a telemetric probe to confirm the phenotype of *Opn3* knockout. To examine the phiC31o integrase-mediated recombination, *Opn3-phiC31o* mice were crossed with the *ROSA26 *^*MultiFPsΔPuro*^ reporter and cyan fluorescent protein, mCerulean, expression was labeled by immunohistochemistry.

**Results:**

The expression pattern of *phiC31o* mRNA was consistent with that of *Opn3* mRNA in *Opn3-phiC31o* heterozygous mouse brains, indicating that *phiC31o* mRNA is expressed under the control of the *Opn3* promoter. Based on the public database, the transcription start site of exon 1 of *Opn3* is identical to that of *Chml*, suggesting that *phiC31o* KI disrupts *Chml* expression. However, *Opn3-phiC31o* homozygous mice sustained *Chml* expression, and the transcription start site of *Chml* was confirmed to be located 112 bp upstream of the predicted second exon. *Opn3-phiC31o* homozygous mice showed a larger decrease in body temperature under cold exposure compared to wild-type controls. In addition, these mice also exhibited a refractive myopia phenotype. These findings confirmed the functional knockout of *Opn3*. Double transgenic mice of *Opn3-phiC31o* and *ROSA26 *^*MultiFPsΔPuro*^ reporter showed mCerulean expression mainly in the olfactory bulb, cerebral cortex, thalamus, and cerebellum. The recombination efficiency was 30% to 44% in the cerebellum.

**Conclusions:**

*Opn3-phiC31o* KI mice were successfully generated. We can generate *Opn3* null mice that does not disrupt *Chml* by preparing homozygotes of *Opn3-phiC31o*. We have deposited the sequences including the newly found transcription start site of *Chml*.

**Supplementary Information:**

The online version contains supplementary material available at 10.1186/s40662-025-00455-z.

## Background

Opn3 is a non-canonical opsin [1] expressed outside the eye and was the first mammalian extraocular opsin to be discovered [2]. Initially identified in the mouse brain, it was originally named encephalopsin [2], and later renamed panopsin after the discovery of its widespread expression in peripheral organs [3]. Opn3 expression was first identified by in situ hybridization (ISH) in the cerebral cortex, striatum, preoptic areas, lateral thalamus, paraventricular nucleus of the hypothalamus, spinal cord, and cerebellar Purkinje cells [2]. Protein expression of Opn3 has also been reported in some parts of the adult mouse brain and retina [4]. The expression of Opn3 in the adult brain and developing nervous system was later examined using Opn3-mCherry (Opn3-mCh) mice [5] and an *Opn3* promoter-driven eGFP (Opn3-eGFP) bacterial artificial chromosome (BAC) transgenic mouse line [6], respectively.

Opn3 is a type of blue light-sensitive G-protein-coupled receptor. Molecular and biochemical properties, such as light sensitivity, retinal binding, G-protein activation, intracellular responses, and absorption spectra, of several Opn3 homologs have been studied [7–11]. Recently, one of the Opn3 homologs, MosOpn3, has been used for optogenetic manipulation in mammals [12], *Caenorhabditis elegans* [13], and zebrafish [14].

Opn3 has been reported to perform a variety of functions, including influencing the intrinsic retinal rhythmic amplitude [15], melanin production in human primary melanocytes [16, 17] and melanocyte survival [18], photorelaxation of smooth muscle in the airway [19] and colon [20], hair growth in the human hair follicle [21], expression in the iris/ciliary body complex [22], and early stages of eye development [23]. In addition, Opn3 is required for the restoration of the barrier function [24], regulation of cancer cell functions [25, 26], and is sensitive to osmotic stimuli and light [27]. *Opn3* knockout mice exhibit a lower body temperature than wild-type mice when challenged with cold conditions [28]. This is because Opn3 is required for blue light enhanced activation of the lipolysis pathway. Other reported phenotypes of *Opn3* knockout include refractive myopia [29], attenuated acoustic startle reflex [30], and susceptibility to diet-induced obesity [31].

Site-specific recombinases (SSRs), such as Cre, are powerful tools for analyzing gene function and tracing the specific progeny of cells that once expressed a gene of interest in vivo [32]. Cre/loxP is the most widely used system for genome manipulation in mice. Recently, several additional SSR systems have been developed, such as Flp/FRT, Dre/rox, and phiC31/att [33, 34]. phiC31 integrases mediate unidirectional recombination between attB and attP sites and produce the resulting recombination sites, attL, and attR [35]. Using transgenic and reporter mice, phiC31 can induce recombination by deleting a stop cassette flanked by attB and attP [36].

The combination of these SSR systems enables simultaneous gene targeting at different gene loci. For a deeper understanding of non-visual opsins in mice, the combination of SSR systems in each opsin gene would be beneficial. While Cre driver lines of *Opn3*, *Opn4*, and *Opn5* exist [28, 37–39], drivers using SSRs other than Cre have not been developed. The aim of this study was to develop *Opn3-phiC31o* knock-in (KI) mice using CRISPR-Cas9 gene editing technology and to validate phiC31 integrase-mediated recombination in heterozygous mice and knockout phenotype in homozygous mice.

## Methods

### Animals

All animal procedures in this study were performed in accordance with the National Institute of Health Guide for the Care and Use of Laboratory Animals and were approved by the Animal Research Committee of Keio University School of Medicine (protocol number: A2023-023). *ROSA26 *^*MultiFPsΔPuro*^ mice [40, 41] were provided by the RIKEN BioResource Research Center (ID: RBRC10966). Both male and female mice were used, as it has been reported that there are no significant differences in Opn3 expression between the sexes [5]. All mice were maintained on a 12/12 h light/dark cycle.

### Generation of *Opn3-phiC31o* KI mice

*Opn3-phiC31o* KI mice were generated using the previously described cloning-free CRISPR-Cas9 system [42]. CRISPR RNA (crRNA) was designed to introduce the double-strand break 7 bp upstream of the endogenous translation start site of *Opn3*. Both crRNA (5'-CCGAGUACAUGGCCCGACGCguuuuagagcuaugcuguuuug-3') and trans-activating CRISPR RNA (tracrRNA, 5'-AAACAGCAUAGCAAGUUAAAAUAAGGCUAGUCCGUUAUCAACUUGAAAAAGUGGCACCGAGUCGGUGCU-3') were chemically synthesized by the manufacturer (Fasmac, Kanagawa, Japan). KI targeting vector with 1.5 kb each of 5' arm and 3' arms as homologous regions and *phiC31o (codon optimized phiC31)-bovine growth hormone poly(A)* sequence was synthesized by Twist Bioscience (South San Francisco, CA, USA). All CRISPR-Cas9 components, including the Cas9 protein (New England Biolabs, Ipswich, MA, USA), cr/tracr RNA, and KI targeting vector, were injected into C57BL/6J (CLEA Japan, Inc., Shizuoka, Japan) fertilized egg at the pronuclei stage. Mice were screened by PCR-based genotyping followed by Sanger sequencing. Founder generation mice were backcrossed to wild-type, and the progenies were used in the experiments.

To detect phiC31o-mediated recombination in adult brain and retina, *Opn3-phiC31o* KI mice were crossed with *ROSA26 *^*MultiFPsΔPuro*^ reporter mice [40]. This reporter strain carries an attB-multiple reporter-attP cassette between the CAG promoter and the *mCerulean* gene. In this study, *Opn3-phiC31o*^+*/*+^ (homozygous); *ROSA26 *^*MultiFPs*^ mice and *Opn3-phiC31o*^+*/−*^ (heterozygous); *ROSA26*^*MultiFPs*^ mice were used to analyze the phiC31o-mediated recombination.

### Genotyping

The following PCR primer sets were used for mouse genotyping: Opn3 IDA F v2 (5'-AGAGGTTCTAAGAAGGCGGA-3') and Opn3 IDA R (5'-GGTGACTCCGAACAGGGATA-3') for the detection of the wild-type allele (742 bp) and the KI allele (2841 bp) using Tks Gflex DNA Polymerase (TAKARA Bio, Shiga, Japan). In some cases, the Opn3 IDA F v2 and Opn3 IDA R primers were used to identify the wild-type allele (742 bp), while the phiC31-F (5'-GAAGCACTTCAGAAAGCAGCAG-3') and Opn3 IDA R primers were used to detect the KI allele (916 bp). These procedures were performed using the KAPA Taq Extra PCR Kit (Kapa Biosystems, Inc., Wilmington, MA, USA).

### Tissue collection

Mice were anesthetized intraperitoneally with a mixture of ketamine (100 mg/kg)-xylazine (10 mg/kg), and then transcardially perfused with 4% paraformaldehyde (PFA)/phosphate buffer solution. The brains were collected and postfixed with 4% PFA overnight at room temperature (RT). After postfixation, brains were cryoprotected in 20% sucrose/phosphate-buffered saline (PBS) until they sank to the bottom of the conical tubes. Brains were snap frozen for approximately 10 s in 2-methylbutane precooled with liquid nitrogen. Coronal sections were cut at 25 µm on a cryostat (Leica CM3050 S, Leica Biosystems, Nussloch, Germany) and mounted on silane-coated glass slides (Matsunami Glass Ind., Ltd., Osaka, Japan). Mouse eyes were also collected after perfusion and postfixed with 4% PFA for 2 h at RT. The eyes were then washed 2–3 times with PBS and the retina was removed from the eye cup for immunohistochemical staining.

### Immunohistochemistry

Brain sections and retinal flatmounts were incubated overnight at RT in a goat polyclonal anti-GFP antibody (1:250, Rockland Immunochemicals Inc., Pottstown, PA, USA). They were then washed and incubated for 2 h at RT in an anti-goat secondary antibody conjugated to Alexa Fluor 488 (1:1000 dilution; Invitrogen, Carlsbad, CA, USA) or Alexa Fluor 555 (1:1000 dilution, Invitrogen). Cerulean is a derivative of GFP and can be detected by the anti-GFP antibody. Fluorescence images were captured using an inverted microscope (BZ-X710, Keyence, Kyoto, Japan) or a confocal microscope (LSM 710, Carl Zeiss AG, BW, Germany). The maximum intensity projection image of a confocal z-stack was obtained using Fiji/ImageJ (National Institutes of Health, Bethesda, MD, USA).

### ISH

ISH was performed as described previously [43]. Briefly, digoxigenin-labeled *phiC31o*, *Opn3,* and *Chml* antisense RNA probes were hybridized to sections, NBT/BCIP (Roche, Basel, Switzerland) was used for color development, and nuclear fast red (Sigma Aldrich, St. Louis, MO, USA) was used for counterstaining. To generate the *Opn3* probe, a plasmid with the same sequence as the *Opn3* probe used in the Allen Brain Atlas (Opn3-RP 050331_04_B02) was synthesized at GenScript Biotech (Piscataway, NJ, USA). To make the *phiC31o* probe, we cloned the phiC31o coding sequence (1815 bp from pPGKPhiC31obpA (addgene plasmid number 13795) into 5' BamHI and 3' EcoRI of pBlueScript II SK ( −) (Agilent Technologies, Inc., Santa Clara, CA, USA). The sense and antisense probe for *Opn3* cover nt 632–1286 (NM_010098; 1763 bp; coding region is 97–1299 bp). The sense and antisense probe for *Chml* probe cover nt 3552–6316 (NM_021350; 6907 bp; coding region is 791–2656 bp).

### Calculation of the recombination efficiency in mice

Multiple areas of the fluorescence images of the cerebellum in which mCerulean expressions were amplified with GFP antibody were selected, and the number of somas with mCerulean expression in the Purkinje cell layer was counted using the cell counter in Fiji/ImageJ (National Institutes of Health). The same regions of the cerebellum in consecutive sections that had undergone ISH with *phiC31o* probe visualization were counted using the same method. The average of the counted areas was taken, and the recombination efficiency in mice was calculated using the following equation:

$$\frac{\text{number of mCerulean }-\text{expressing soma}}{\text{number of }phiC31o-\text{expressing cells}}\times 100\%$$

### Measurement of body temperature using telemetric probes

Telemetric probes (Anipill, BodyCAP, Hérouville Saint-Clair, France) were surgically implanted intraperitoneally in mice under ketamine/xylazine anesthesia. Mice were allowed to recover for at least 5 days after surgery. Mice were then placed in a 4 °C chamber (BR-33FL, TAITEC, Saitama, Japan) and body temperature was recorded every 5 min for 90 min. Male and female mice aged 2- to 7-month-old were used. Food and water were provided ad libitum during the cold treatments.

### Measurement of ocular biometric parameters

Prior to measurements, 0.5% tropicamide and 0.5% phenylephrine eye drops (Santen Pharmaceutical Co., Ltd., Osaka, Japan) were administered to induce mydriasis. Then, mice were anaesthetized via intraperitoneal injection of a mixture containing midazolam, medetomidine, and butorphanol tartrate (0.01 mL/g bodyweight).

Refractive status was assessed using an infrared photorefractor (Steinbeis Transfer Center, Graz, Austria). Axial length was measured with a spectral-domain optical coherence tomography (SD-OCT) system (Envisu R4310; Leica, Wetzlar, Germany) and was defined as the distance between the corneal vertex and the retinal pigment epithelium layer near the optic nerve head. Anterior chamber depth, lens thickness, and vitreous chamber depth were calculated based on the proportional lengths of each region measured on OCT scans using ImageJ (NIH), normalized to the total axial length. For choroidal analysis, an OCT image was acquired from a retinochoroidal region located 500 µm from the optic nerve head. The area between the retinal pigment epithelium and the posterior choroidal boundary was quantified using ImageJ and the choroidal thickness (ChT) was calculated as the ratio of this area to the corresponding boundary length, as previously described [44]. Corneal radius of curvature was measured using a separate infrared photorefractor (Steinbeis Transfer Center, Germany) according to a previously described method [45]. After general anesthesia and mydriasis, eight infrared LEDs arranged in a circular pattern illuminated the mouse cornea, and reflected light was captured by an infrared camera. The diameter of the reflected light ring on the corneal surface was used to determine corneal curvature.

### 5' Rapid amplification of cDNA end (5' RACE) analysis

Experiments were performed using the GeneRacer kit according to the manufacturer's instructions (ThermoFisher Scientific, Waltham, MA, USA). Total RNA was isolated from the cerebral cortex and cerebellum of a 3-month-old male C57BL/6J mouse using Ambion TRIzol Reagent (Life Technologies, Carlsbad, CA, USA). Five micrograms of total RNA were reverse transcribed using the GeneRacer kit with SuperScript III RT and GeneRacer Oligo dT Primer. Amplification of 5' cDNA ends was performed using Tks Gflex DNA Polymerase (TAKARA Bio) with the GeneRacer 5' Primer and a *Chml* gene-specific primer (5'-TGCTTCACCACAGGTTCCAAGTCTTC-3') in the first round of PCR. Nested PCR was performed using the GeneRacer 5' Nested Primer and a *Chml* gene-specific nested primer (5'-CCATGACCCGAACAGAAGTGGTTCC-3'). Gel electrophoresis of the 5' nested PCR products yielded a single band of approximately 2 kb size in both the cerebral cortex and cerebellum. These PCR fragments were cloned using the Zero Blunt TOPO PCR Cloning Kit for Sequencing (Invitrogen) and sequenced (Eurofins Genomics, Tokyo, Japan). The cloned sequence was submitted to the EMBL/GenBank/DDBJ database (accession number LC816633).

### Statistical analysis

Statistical analyses were performed using Python 3.9. The graph showing the decrease in body temperature in the cold exposure experiment was analyzed by one-way repeated measures ANOVA. The level of significance was defined as a *P* value of less than 0.05. Data are presented as mean ± standard error.

## Results

### Generation and validation of *Opn3-phiC31o* KI mice

**Fig. 1:**
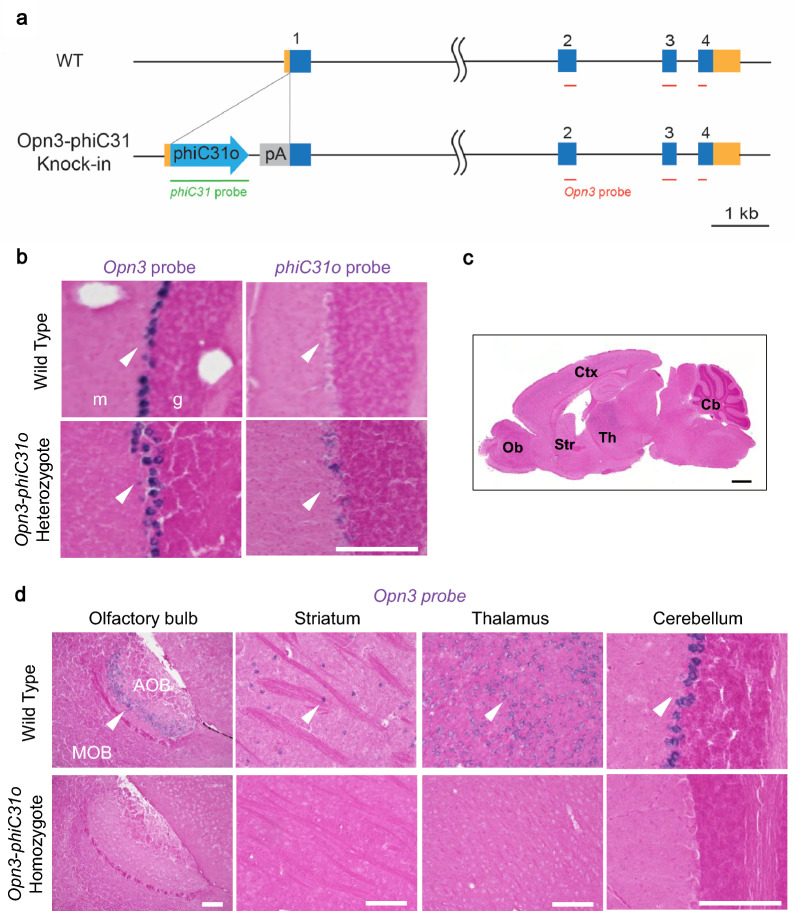
Generation of *Opn3-phiC31o* KI mice using CRISPR-Cas9. **a** Schematic representation of the wild-type allele of the *Opn3* gene according to the NCBI genome annotation (NM_010098.4) and the KI allele, *Opn3-phiC31o.* **b** Validation of *Opn3-phiC31o* expression using the heterozygous *Opn3-phiC31o* mouse. ISH results for *Opn3* mRNA and *phiC31o* mRNA are shown in representative coronal sections of the cerebellum of the heterozygous *Opn3-phiC31o* KI mouse. g, granule layer; m, molecular layer. Scale bar: 150 μm. **c** A schematic image of the sagittal brain highlighting regions examined in this paper. Ob, olfactory bulb; Ctx, cortex; Str, striatum; Th, thalamus; Cb, cerebellum. Scale bar: 1 mm. **d** Validation of *Opn3* knockout in homozygous *Opn3-phiC31o* mice. Representative sagittal sections of the olfactory bulb, striatum, thalamus and cerebellum from wild-type and homozygous mice were subjected to ISH using the *Opn3* probe. *Opn3* mRNA expression in the *Opn3-phiC31o* homozygous mouse was then compared with that in the wild-type mouse. *AOB,* accessory olfactory bulb; *MOB,* main olfactory bulb. Scale bars: 150 µm

We generated KI mice in which *phiC31o-poly(A)* was inserted into the translation start site of *Opn3* using CRISPR-Cas9 genome editing (Fig. 1a). To confirm whether *phiC31o* is expressed under the control of the *Opn3* promoter, and whether the *phiC31o* mRNA expression pattern reflects the endogenous *Opn3* mRNA expression, ISH with *Opn3* and *phiC31o* probes was performed in serial sections of *Opn3-phiC31o* heterozygous mouse cerebellum. The cerebellum was selected for comparison due to its well established high *Opn3* expression in the brain [5]. In two consecutive brain sections, endogenous *Opn3* and exogenous *phiC31o* signals were similarly localized in the Purkinje cell layer of the cerebellum (Fig. 1b), with *phiC31o* reflecting approximately 97% of *Opn3* expression. This suggests that the expression of *phiC31o* in the *Opn3-phiC31o* mouse line corresponds to the endogenous *Opn3* expression. *Opn3* signal levels were not observed in *Opn3-phiC31o* homozygous mouse samples (Fig. 1c, 1d), indicating the successful knockout of *Opn3*.

### *Opn3* was functionally knocked out in our *Opn3-phiC31o* mice

**Fig. 2:**
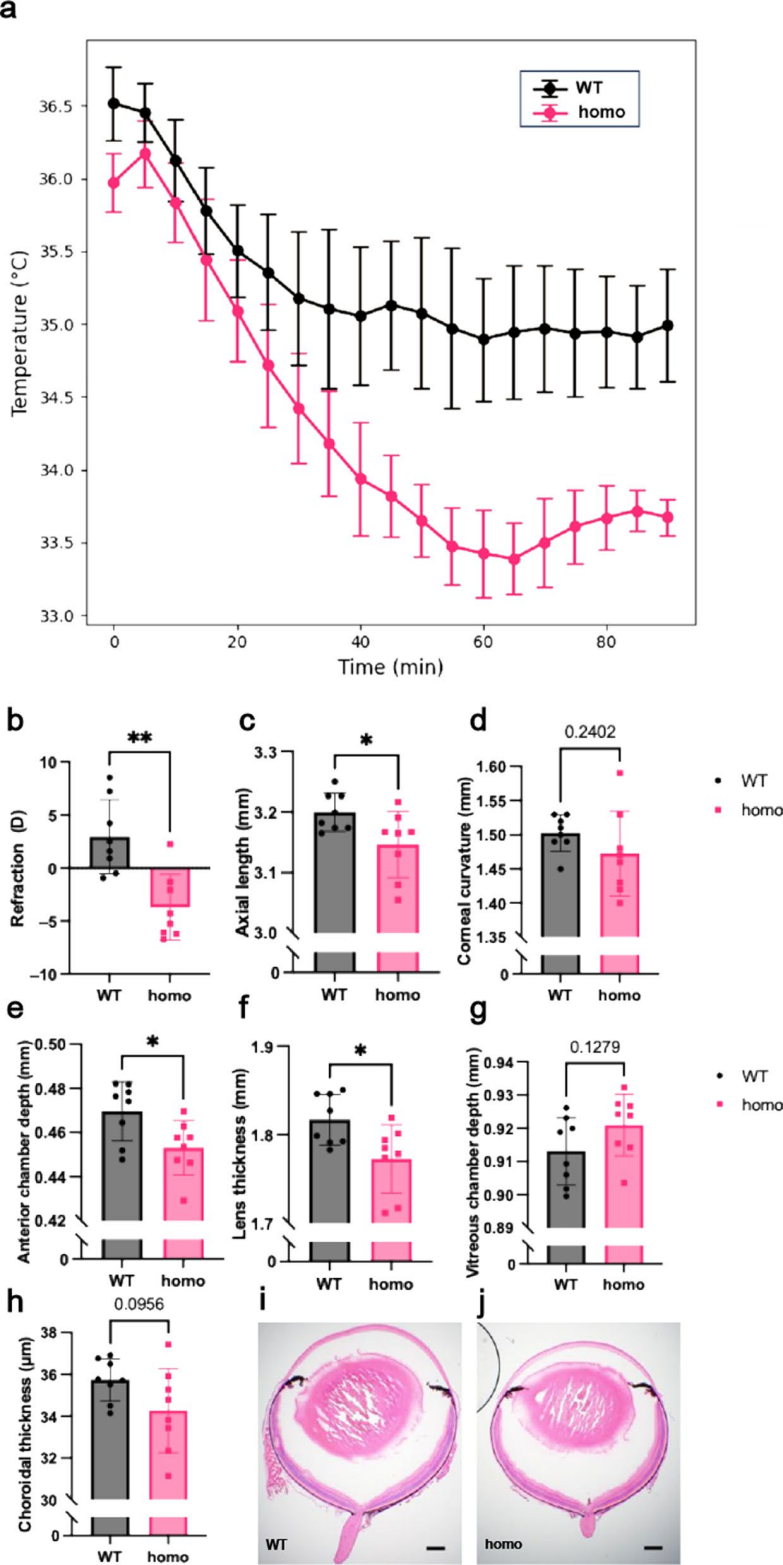
*Opn3* knockout functional experiment in homozygous *Opn3-phiC31o* mice. **a** Decrease in body temperatures of wild-type (n = 5) and *Opn3* knockout mice (n = 5) after cold exposure (*P* = 0.044; One-way repeated measures ANOVA). Body temperatures were recorded every 5 min for 90 min. Data is presented as mean ± standard error. WT, wild-type mice; homo, *Opn3-phiC31o* homozygous mice. **b** Refraction; **c** Axial length; **d** Corneal radius of curvature; **e** Anterior chamber depth; **f** Lens thickness; **g** Vitreous chamber depth; **h** Choroidal thickness. All parameters were measured in wild-type littermates (n = 8) and homozygous *Opn3-phiC31o* mice (n = 8). Data are presented as mean ± standard deviation. Statistical comparisons were performed using a two-tailed unpaired t-test with Welch’s correction. *P < 0.05; **P < 0.01. **i** Representative frozen sections of wild-type eyeballs (H&E staining). Scale bar: 200 µm. **j** Representative frozen sections of homo eyeballs (H&E staining). Scale bar: 200 µm

Previous studies have reported that *Opn3* knockout mice exhibit a significantly larger decrease in body temperature than wild-type mice in response to cold exposure [28]. We recreated these cold exposure experiments to functionally validate the *Opn3-phiC31o* allele. *Opn3-phiC31o* homozygous mice (n = 5) and wild-type mice (n = 5) were both surgically implanted with a telemetric probe under general anesthesia and subsequently placed in a 4 °C chamber. The body temperatures of both groups were recorded every 5 min over a 90 min period. Analysis using one-way repeated measures ANOVA revealed that the body temperature of the *Opn3* knockout mice was significantly lower than that of the wild-type mice (*P* = 0.044, Fig. 2a). This result is consistent with previous findings.

To further confirm the functional knockout of *Opn3*, we assessed refractive measurements and ocular biometry in *Opn3-phiC31o* homozygous mice. Refractive assessments revealed that *Opn3-phiC31o* homozygous mice (n = 8) exhibited a myopic phenotype compared to wild-type controls (n = 8, Fig. 2b).

Ocular biometry was performed using a SD-OCT system. Axial length (Fig. 2c), anterior chamber depth (Fig. 2e), lens thickness (Fig. 2f), vitreous chamber depth (Fig. 2g), and choroidal thickness (Fig. 2h) was measured. In *Opn3-phiC31o* homozygous mice, axial length was significantly reduced (Fig. 2c), along with decreased anterior chamber depth (Fig. 2e) and lens thickness (Fig. 2f), compared to wild-type mice. No significant differences were observed in vitreous chamber depth (Fig. 2g) or choroidal thickness (Fig. 2h). In addition, corneal curvature did not differ significantly between groups (Fig. 2d). These results are consistent with previous reports that *Opn3* knockout mice exhibit a myopia-like phenotype [29], and further support the successful functional knockout of *Opn3* in our mouse model.

### The *Opn3-phiC31o* homozygotes did not disrupt *Chml* gene transcription

**Fig. 3:**
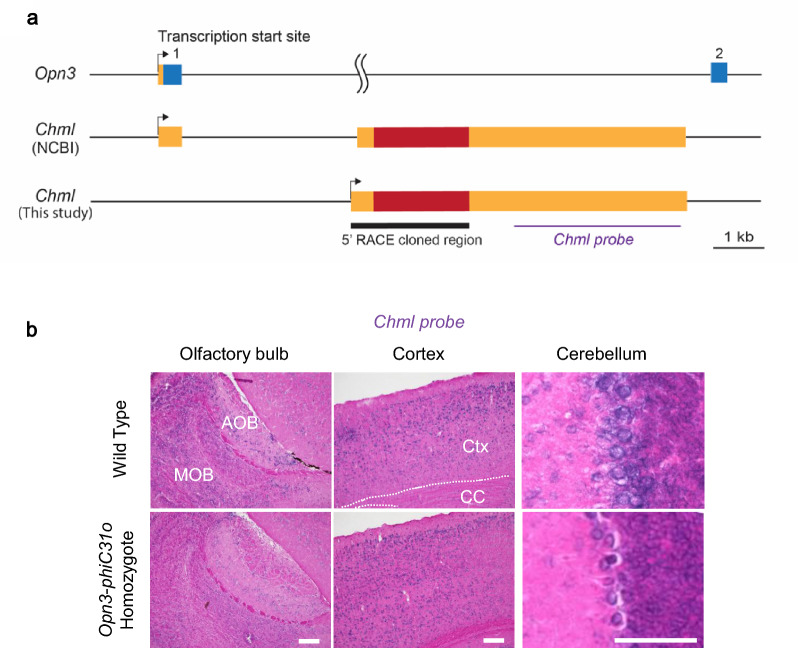
The transcription start site of *Chml* was different from the NCBI genome annotation. **a** Schematic diagram of the *Opn3* gene (NM_010098.4) and the *Chml* gene (NM_021350.3) according to the NCBI genome annotation, as well as the *Chml* gene structure cloned by 5' RACE analysis using RNA from the cerebral cortex and cerebellum. **b** Representative sagittal sections of the olfactory bulb, cortex, and cerebellum showing *Chml* expression in wild-type and *Opn3-phiC31o* homozygous mice. *AOB,* accessory olfactory bulb; *MOB,* main olfactory bulb; *Ctx,* cortex; *CC,* corpus callosum. Scale bars: 150 µm

*Chml*, also known as *choroideremia-like*, is a gene associated with diseases such as choroideremia [46]. According to the National Center for Biotechnology Information (NCBI) genome annotation, the first exon of *Chml* overlaps with the first exon of *Opn3* (Fig. 3a**)**. Therefore, knocking in of *phiC31o-poly(A)* at the first exon of *Opn3* is predicted to disrupt both *Opn3* and *Chml* genes. However, ISH analysis of *Chml* expression in the olfactory bulb, cortex, and cerebellum in both wild-type and *Opn3-phiC31o* homozygous mice still showed *Chml* signals in the latter (Fig. 3b), demonstrating that *Chml* is not disrupted in the *Opn3-phiC31o* homozygous mouse. To determine the exact transcription start site of *Chml*, RNA was isolated from the cerebral cortex and cerebellum and analyzed by the 5' RACE technique (see Methods). Sequence analysis of 11 clones from the cerebral cortex and 6 clones from the cerebellum by 5' RACE showed that they all have identical sequences (EMBL/GenBank/DDBJ accession number LC816633) and that, at least in the cerebral cortex and cerebellum, the transcribed region of *Chml* did not initiate at the predicted first exon but starts transcription at 112 bp upstream of the predicted second exon (Fig. 3a), confirming that our mouse line is a single knockout model of *Opn3*.

### Recombination efficiency of the *Opn3-phiC31o* mice is low

**Fig. 4:**
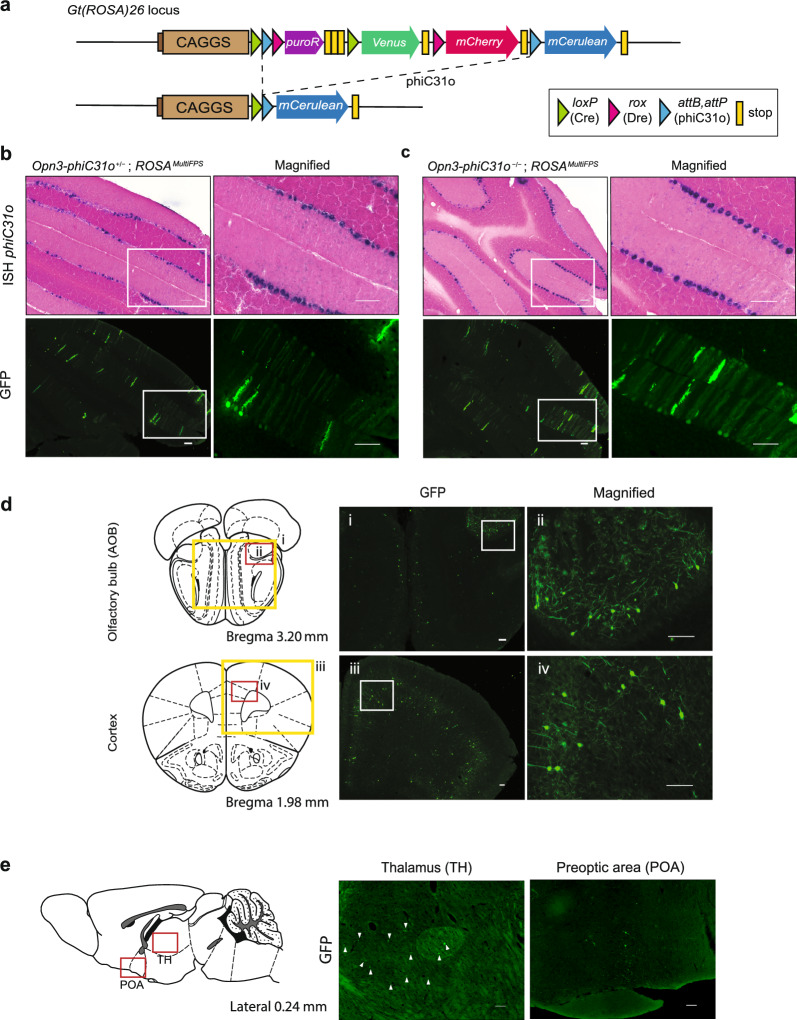
Recombination efficiency and Opn3 expression in *Opn3-phiC31o* KI mice brain. **a** Schematic representation of the *ROSA26 *^*MultiFPs*^ allele [40]. Multiple fluorescent proteins are inserted into the allele at the Gt(ROSA)26 locus under the control of the ubiquitous CAGGS promoter. mCerulean is activated by phiC31o-specific recombinase in response to the *Opn3-phiC31o* gene expression. **b** Representative coronal sections of the cerebellum for the calculation of recombination efficiency in the *Opn3-phiC31o*^+*/−*^; *ROSA26 *^*MultiFPs*^ mouse. Magnified images of the cerebellum with visualization of *phiC31o* probe and GFP expression are shown. Scale bars: 100 μm. **c** Representative coronal sections of the cerebellum showing the recombination efficiency in *Opn3-phiC31o*^+*/*+^; *ROSA26 *^*MultiFPs*^ mouse. Magnified images of the cerebellum with visualization of the *phiC31o* probe and GFP expression are shown. Scale bars: 100 μm. **d** Representative coronal sections of phiC31o-mediated mCerulean expression amplified with GFP antibody in the olfactory bulb (i) and the cerebral cortex (iii). Magnified images of the boxed regions are shown (ii, iv). Scale bars: 100 µm. **e** Representative sagittal sections of the brain of the *Opn3-phiC31o*^+*/*+^; *ROSA26 *^*MultiFPs*^ mouse showing phiC31o-mediated mCerulean expression amplified with GFP antibody in the thalamus (TH) (labelled with arrowheads) and preoptic area (POA). Scale bars: 100 µm

To detect phiC31o-mediated recombination in adult brains, we crossed *Opn3-phiC31o* KI mice with *ROSA26 *^*MultiFPsΔPuro*^ reporter mice [40]. This reporter strain carries an attB-multiple reporter-attP cassette between the CAG promoter and the *mCerulean* gene (Fig. 4a**)**. Upon recombination mediated by phiC31o, the mCerulean cyan fluorescent protein will be expressed. To amplify the mCerulean reporter signal, brain sections were immunostained with an anti-GFP antibody. We then evaluated the recombination efficiency of phiC31o using serial sections of *phiC31o* ISH probe and GFP antibody. By counting the *phiC31o*-positive signals and GFP-positive signals in the consecutive section, we estimated that the phiC31o-mediated recombination in the cerebellum of *Opn3-phiC31o*^+*/−*^; *ROSA26 *^*MultiFPs*^ mice occurred at 30.3% ± 6.36% (n = 3), while that of *Opn3-phiC31o*^+*/*+^; *ROSA26 *^*MultiFPs*^ mice was 44.4% ± 11.8% (n = 3). These data demonstrated that the expression of mCerulean reporter in the cerebellum of the mice heterozygous for the *Opn3-phiC31o* KI allele, *Opn3-phiC31o*^+*/−*^; *ROSA26 *^*MultiFPs*^ (Fig. 4b), was lower than that in the cerebellum of the mice homozygous for *Opn3-phiC31o* KI allele, *Opn3-phiC31o*^+*/*+^; *ROSA26 *^*MultiFPs*^ (Fig. 4c).

*Opn3*-phiC31-mediated mCerulean expression was examined in various regions of the mouse brain (Table 1). We observed mCerulean expression in the olfactory bulbs (Fig. 4d), cerebral cortex (Fig. 4d), Purkinje cell layer of the cerebellum (Fig. 4b, c), and slightly in the thalamus (Fig. 4e) and preoptic area (Fig. 4e). These reporter protein expression patterns are included in previously published reporter lines [5, 6].

**Table 1:** phiC31-mediated gene expression in *Opn3-phiC31o; ROSA26 *^*MultiFPs*^ mice

Brain region	Expression-positive structures
Cortex	ACA, AI, AUD, MO, PTLp, RSP, ORB, SS, VIS
Olfactory areas	AOB, MOB
Striatum	Not observed
Nucleus accumbens	Not observed
Hippocampal formation	Not observed
Thalamus	Subregions not identified
Hypothalamus	POA
Cerebellum	DN, FN, IP, Purkinje
Midbrain	Not observed
Pons	Not observed
Medulla	Not observed
Retina	HC, RGC

phiC31-mediated gene expressing CNS regions were shown in bold. The brain region list was created based on previous literature [5]. Cortex: *ACA = *anterior cingulate area; *AI* = agranular insular area; *AUD* = auditory area; *MO* = somatomotor area; *PTLp =* posterior parietal association area; *RSP =* retrosplenial area; *ORB* = orbital area;* SS* = somatosensory area; *VIS =* visual area; Olfactory areas: *AOB =* accessory olfactory bulb; *MOB* = main olfactory bulb. Hypothalamus: *POA =* preoptic area. Cerebellum: *DN* = dentate nucleus; *FN* = fastigial nucleus; *IP =* interposed nucleus. Retina: *HC =* horizontal cell; *RGC =* retinal ganglion cell

### Low recombination efficiency in the retina

**Fig. 5:**
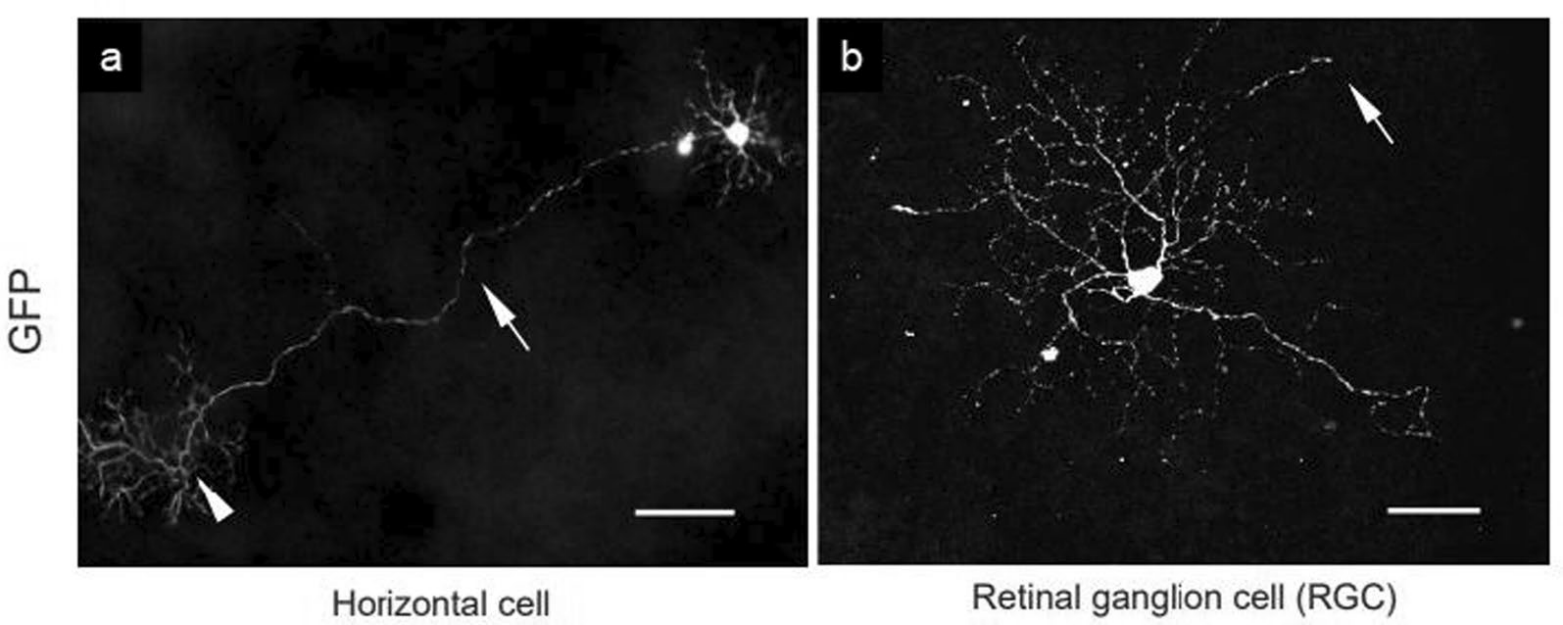
mCerulean-expressing cells in *Opn3-phiC31o* KI mice retina despite the low recombination efficiency. phiC31o-mediated mCerulean expression labelled with GFP antibody in the retina of *Opn3-phiC31o*^+*/*+^; *ROSA26 *^*MultiFPs*^ mice. A small number of (**a**) horizontal cells (HCs) and (**b**) retinal ganglion cells (RGCs) showed mCerulean expression. The HC and RGC were imaged by confocal and fluorescence microscope, respectively. **a** Arrow and arrowhead indicate an axon and axon terminal arborization of the HC, respectively. **b** Arrow indicates an axon of the RGC. Scale bars: 50 µm.

In addition to the phiC31o-mediated recombination in the brain, we examined recombination in the retina. Retinas were stained with GFP antibody to amplify the mCerulean signals and observed under a fluorescence microscope. However, recombination in the retina was inconsistent, showing extremely low or no occurrence. Among the 12 retinas (n = 6 mice) that we observed, only 3 of the retinas contained 5 mCerulean-expressing cells [1 horizontal cell (HC) from 1 retina (Fig. 5a) or 4 retinal ganglion cells (RGCs) from 2 retinas (Fig. 5b)].

## Discussion

Since the functional role of Opn3 is the least studied among the non-visual opsins [1], its expression in mammals has been extensively studied in recent years. It has been reported that *Opn3* mRNA expression is found not only in the central nervous system (CNS), but also in peripheral tissues such as adipose tissue [28], skin [5], smooth muscles [19], and blood vessels [47]. Since there are currently no reliable antibodies for murine Opn3, several mouse reporter lines have been developed to assess *Opn3* promoter activity, including *Opn3-mCh* KI [5] and *Opn3*-eGFP BAC transgenic mice [6]. To further investigate *Opn3* expression in the CNS, we generated a new KI mouse model, *Opn3-phiC31o*.

Codon-optimization of the *phiC31* gene into *phiC31o* has been reported to significantly improve recombination activity in embryonic stem (ES) cells, to a level similar to that of Cre recombinase [34]. However, the recombination efficiency of phiC31o was later reported to be lower than that of Cre- or Flpe-expressing transgenic mice [36]. For example, the phiC31o-mediated recombination efficiency of TH-PhiC31o mice crossed with the R26attR reporter has been reported to range from 4.7% to 48.2% in various brain regions [36]. The recombination efficiency of phiC31o was also observed to be relatively low compared with Cre and Dre in a recent study, which was around 76% [40]. Consistent with these findings, our *Opn3-phiC31o*^+*/−*^; *ROSA26 *^*MultiFPs*^ showed ~ 30% recombination efficiency in the Purkinje cell layer of the cerebellum.

Previous research using *Opn3-mCh* mice revealed additional brain regions with Opn-mCh expression such as the mitral cell layer, the posterior hypothalamic nucleus, and the superior olivary complex in the pons [5]. However, in our *Opn3-phiC31o* mice, we observed only limited signals in the brain, mainly in the olfactory bulb, cerebral cortex, and the Purkinje cell layer of the cerebellum. It has also been reported that Opn3-mCh expression is abundant in hypothalamic formation, but in our *Opn3-phiC31o* mice, we observed only sparse signals in the preoptic area within sagittal brain sections. Moreover, although Opn3-mCh was reported to be most highly expressed in the thalamus, our model showed limited signals in the thalamus in sagittal brain sections. The advantage of the Opn3-mCh reporter mouse over other models is that the Opn3-mCherry fusion protein is expressed under the native promoter, thereby preserving endogenous levels of Opn3 with native expression throughout development [5]. This is important for further characterization of Opn3, as Opn3 is developmentally regulated, at least in the brain [2, 6, 48].

Opn3 expression in the developing nervous system has also been examined in previous studies using an *Opn3* promoter-driven eGFP BAC transgenic mouse line [6]. It has been reported that Opn3-eGFP was detected in 25 new structures in the central and peripheral nervous system that were not previously reported to have Opn3 expression, including sensory ganglia, the olfactory epithelium, vestibular nuclei, and parafascicular nucleus [6]. Nevertheless, the regions of Opn3 expression reported in those studies were much broader than what we observed in *Opn3-phiC31o*; *ROSA26 *^*MultiFPsΔPuro*^ mice. Knowing that BAC-based GFP reporters have the possibility to be leaky and generate false positives [5], this discrepancy once again proves that the recombination efficiency in our newly generated mouse line was not satisfactory enough to thoroughly examine Opn3 expression in the CNS.

Opn3-eGFP has previously been reported to be expressed in RGCs [29], and thus we examined *Opn3-phiC31o*-mediated gene expression in the retina of our mouse line. We found extremely low recombination efficiency in the retina of the *Opn3-phiC31o*; *ROSA26 *^*MultiFPsΔPuro*^ mice. Although *Opn3* promoter activity is observed throughout the ganglion cell layer at birth, the proportion of RGCs expressing Opn3 in the adult brain decreases and is restricted to a few cells, as reported in previous studies [29]. Since phiC31o-mediated recombination is therefore more likely to occur at birth, and mCerulean expression should be indelible, our results suggest that the recombination efficacy of phiC31o is insufficient to conduct recombination even at the neonatal stage. Previous studies have also shown that approximately 24% of Opn3-eGFP-positive RGCs co-express Opn4 [29]. Opn4-positive RGCs are categorized into M1 to M5 subtypes based on morphological characteristics, with M2 and M4 subtypes known to express Opn3-eGFP [49]. Therefore, sparse labelling through Opn3-phiC31o-mediated recombination may allow identification of RGCs co-expressing Opn4, especially M2 and M4 types. If a sufficient number of labeled RGCs are recovered for morphological analysis, this could potentially enable morphological characterization of these specific RGC populations.

Despite the low recombination efficiency of our mouse line, one of the positive achievements is the observation of cell morphological structure. Due to the low recombination efficiency, there are few mCerulean-expressing cells which allow us to easily observe the whole morphological structure of the RGCs and HCs. Mice have only one type of HC, called the B-type axon-bearing HC [50, 51], which shows the same morphological structure as the one stained in our slides. Although the number of HCs we discovered was severely low, which may raise doubt about the reliability of Opn3 expression in HCs, an Opn3 homolog, cTMT, has been reported to be expressed in chicken retinal HCs [8]. This provides evidence that Opn3 expression can be found in retinal HCs, and thus there is a high possibility that it is also expressed in other animals, such as mice in this case. Nevertheless, it should be noted that phiC31o-mediated recombination in the retina was extremely few and the number of labeled RGCs and HCs was limited. Further studies are needed to confirm the Opn3 expression pattern, as well as how these Opn3-expressing neurons respond to blue light and modulate intracellular signaling in an Opn3-dependent manner. However, it would be necessary to perform experiments using a system in which more RGCs and HCs can be labeled in the retina, rather than the *Opn3-phiC31o*; *ROSA26 *^*MultiFPsΔPuro*^ mice reported here.

With the generation of the *Opn3-phiC31o* mouse line, a future approach would be to generate an *Opn5-Dre* mouse line, and then to cross these two mouse lines with the existing *Opn4-Cre* mouse line [38, 39] and the *ROSA26 *^*MultiFPsΔPuro*^ reporter mice [40]. By crossing these 4 mouse lines, the expressions of the non-visual opsins, Opn3, Opn4, and Opn5, could be studied simultaneously within the same mouse individual, since phiC31o, Cre, and Dre drivers mediate the recombination and express different fluorescent proteins, mCerulean, Venus, and mCherry, respectively, as reported previously [40].

There are several limitations in this experiment that should be considered in future studies, which may arise from technical aspects of the procedures. First, in the analysis of the transcription start site of *Chml* gene using the 5' RACE method, we used clones derived from the cerebral cortex and cerebellum of a single 3-month-old male C57BL/6 J mouse. We did not confirm whether the same transcription start site of *Chml* could be consistently identified across multiple animals. Next, the recombination efficiency of phiC31o was extremely low, resulting in very sparse mCerulean labeling in *Opn3-phiC31o*^+*/*+^*; ROSA *^*MultiFPS*^ mice. Although we made every effort to analyze the brain as comprehensively as possible, it is possible that we failed to detect all the sparsely distributed recombination-positive cells. The absence of mCerulean-positive cells in the paraventricular nucleus may be attributable to this limitation. To more comprehensively visualize mCerulean-positive cells, the use of tissue clearing techniques may have been beneficial.

## Conclusions

We have developed an *Opn3*-*phiC31o* KI mouse line. Although *phiC31o* exhibits low recombination efficiency in the brain and retina, this line serves as a valuable resource for the random and sparse labelling of individual cells, facilitating detailed morphological characterization. Additionally, it can function as an *Opn3* null model without perturbing *Chml*, thereby expanding its utility for diverse experimental applications.

## Supplementary Information

Supplementary Figure 1. In situ hybridization results using sense probe showed no signals in brain sections. Representative sagittal sections of the cerebellum and thalamus from wild-type and Opn3-phiC31o homozygous mice were subjected to ISH using the Opn3 sense probe, phiC31o sense probe, and Chml sense probe. Opn3 mRNA expression in the Opn3-phiC31o homozygous mouse brain was not detected. Scale bars: 300 µm.

## Data Availability

All data generated or analyzed during this study are included in this published article, and EMBL/GenBank/DDBJ (accession number LC816633).

## Abbreviations

BAC: Bacterial artificial chromosome
crRNA: CRISPR RNA
CNS: Central nervous system
HC: Horizontal cell
ISH: In situ hybridization
KI: Knock-in
NCBI: National Center for Biotechnology Information
PBS: Phosphate-buffered saline
PFA: Paraformaldehyde
RGC: Retinal ganglion cell
RT: Room temperature
SSR: Site-specific recombinase
tracrRNA: Trans-activating CRISPR RNA
5' RACE: 5' Rapid amplification of cDNA end
