# Supplementary figures and images for: Validation of phiC31-mediated expression and functional knockout of Opn3 in the Opn3-phiC31o knock-in mouse
Source: Eye Vis (Lond). 2025 Oct 17;12:41. doi: 10.1186/s40662-025-00455-z (PMC12532464; doi:10.1186/s40662-025-00455-z)

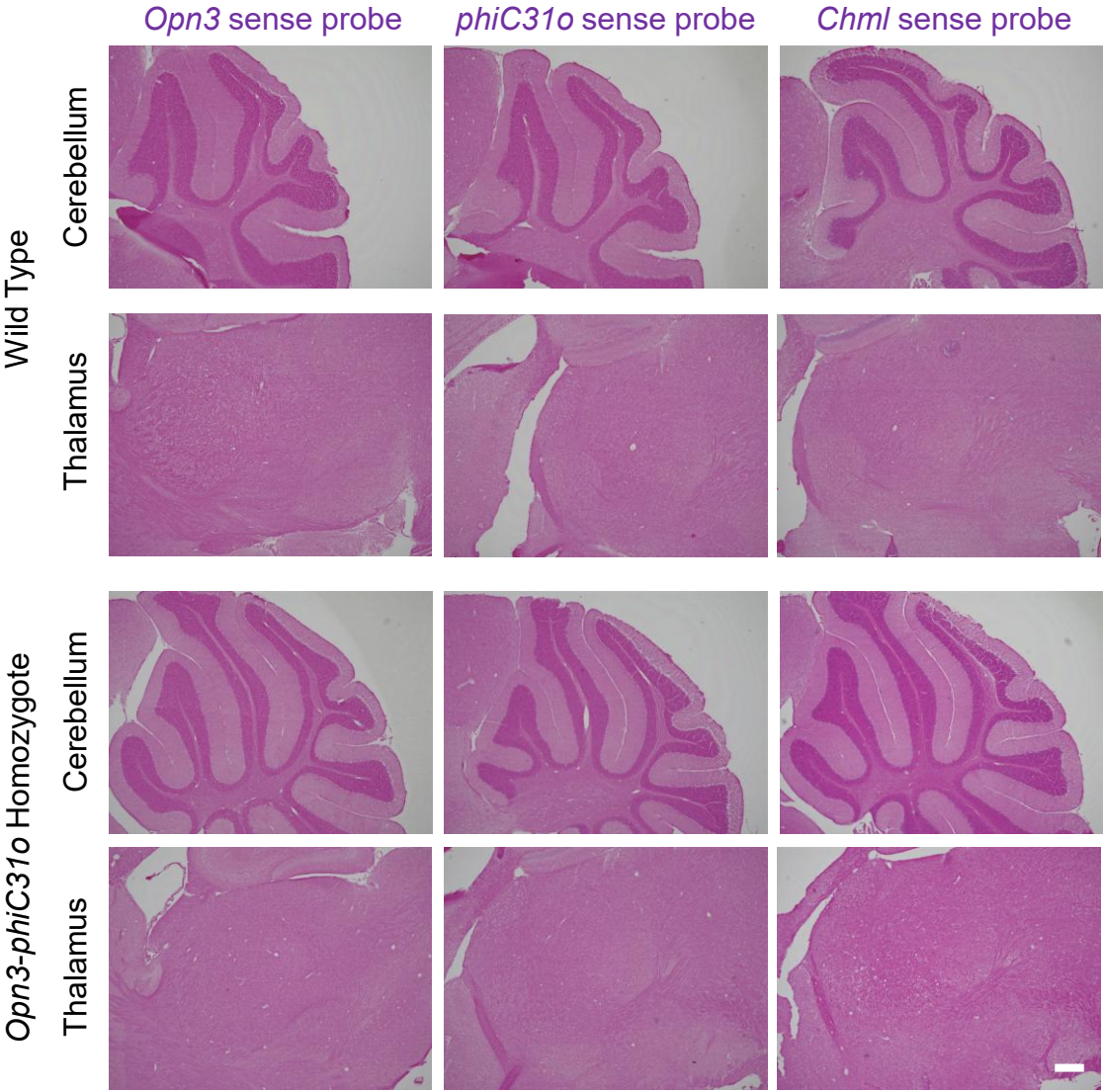

Supplement: Supplementary file 1 — Supplementary Figure 1. In situ hybridization results using sense probe showed no signals in brain sections. Representative sagittal sections of the cerebellum and thalamus from wild-type and Opn3-phiC31o homozygous mice were subjected to ISH using the Opn3 sense probe, phiC31o sense probe, and Chml sense probe. Opn3 mRNA expression in the Opn3-phiC31o homozygous mouse brain was not detected. Scale bars: 300 µm. [file 40662_2025_455_MOESM1_ESM.pdf]
